# Infection as an Environmental Trigger of Multiple Sclerosis Disease Exacerbation

**DOI:** 10.3389/fimmu.2015.00520

**Published:** 2015-10-19

**Authors:** Andrew J. Steelman

**Affiliations:** ^1^Department of Animal Sciences, College of Agricultural, Consumer and Environmental Sciences, University of Illinois Urbana-Champaign, Urbana, IL, USA; ^2^Neuroscience Program, University of Illinois Urbana-Champaign, Urbana, IL, USA; ^3^Division of Nutritional Sciences, University of Illinois Urbana-Champaign, Urbana, IL, USA

**Keywords:** multiple sclerosis, neuroinflammation, relapse, viral infection, natural history

## Abstract

Over the past several decades, significant advances have been made in identifying factors that contribute to the pathogenesis of multiple sclerosis (MS) and have culminated in the approval of some effective therapeutic strategies for disease intervention. However, the mechanisms by which environmental factors, such as infection, contribute to the pathogenesis and/or symptom exacerbation remain to be fully elucidated. Relapse frequency in MS patients contributes to neurological impairment and, in the initial phases of disease, serves as a predictor of poor disease prognosis. The purpose of this review is to examine the evidence that supports a role for peripheral infection in modulating the natural history of this disease. Evidence supporting a role for infection in promoting exacerbation in animal models of MS is also reviewed. Finally, a few mechanisms by which infection may exacerbate symptoms of MS and other neurological diseases are discussed. Those who comprise the majority of MS patients acquire approximately two upper-respiratory infections per year; furthermore, this type of infection doubles the risk for MS relapse, underscoring the contribution of this relationship as being potentially important and particularly detrimental.

## Multiple Sclerosis

Multiple sclerosis (MS) is a relatively prominent autoimmune disease identified as the number one cause of non-traumatic adult onset neurological disability (1). Moreover, MS is particularly cruel insofar as its peak age of onset occurs at a relatively early stage in life and, over time, transitions to a progressive disease course that ultimately leads to a loss of neurological function (1). In fact, most MS patients experience symptoms that include, but are certainly not limited to, decreased mobility, vision, and cognitive function as well as increased anxiety, depression, and fatigue. It has been estimated that MS incurs an annual cost of $6.8 billion, which amounts to approximately $40,000 per affected individual. Roughly, 400,000 people are affected by this disease in the US alone, and its incidence may be increasing (2, 3).

While the cause of MS remains unknown, this disease is thought to be attributable to an autoreactive attack on myelin and oligodendrocytes by cells of the immune system that have entered the brain and/or spinal cord. Results from genome-wide association studies (4, 5) as well as the effectiveness of treatment strategies that directly target the activation or trafficking of T-cells and B-cells into the CNS (6–9) lend strong support for such a hypothesis.

Roughly, 8% of all MS patients exhibit a relapsing-remitting disease course that is characterized by bouts of clinical disability and clinical remission that are separated by time (1). While a great deal of research has been focused on mechanisms governing immunopathology during relapses, much less research has addressed the environmental or physiological factors that underlie the evolution of clinical relapse. Understanding both the environmental triggers that contribute to relapses as well as the biological pathways by which immune cells are attracted to the CNS, become activated, and contribute to neuroinflammatory processes is pertinent to the development of therapeutics, both pharmacological and interventional, that can reduce relapses and neurodegeneration. Moreover, comprehending the basic physiology underlying bidirectional communication pathways between the brain and the immune system will likely uncover potential mechanisms for intervention for a myriad of inflammatory demyelinating and neurodegenerative diseases.

## Infection and Disease Exacerbation in MS Patients

### Influence of Seasons

The earliest evidence that demonstrates a role for upper-respiratory infections in the promotion of MS exacerbation is derived from a study performed by Sibley and Foley that was published nearly 40 years ago (10). While this long-term prospective study comprised only a few patients, the authors were able to make several pertinent observations. First, increased serum antibody titers to measles, respiratory syncytial, adenovirus, herpes simplex, or mumps were not associated with either MS onset or exacerbation. Second, they did not demonstrate a correlation between vaccination (Influenza, poliovirus, measles) and MS exacerbation. Instead, the authors identified a critical link between MS exacerbation and infection. Specifically, it was noted that a large proportion of MS exacerbations were associated with infection, that is, that exacerbations occurred within 1 week prior to and 5 weeks after the appearance of symptoms typical of ­infection – including coryza, sore throat, cough, and diarrhea. Finally, they showed that the predilection for relapse is seasonal with peak times occurring between the months of March and April, then again between July and September.

A link between seasons and increased relapse risk was subsequently described by other groups conducting independent studies in multiple countries including the US (11), Switzerland (12), and Japan (13), and was further established by data that demonstrated that seasonal transitions were associated with fluctuations in gadolinium-enhancing MRI activity in MS patients (14). While not all studies reported that MS relapse is dependent on seasonality, a meta-analysis study by Jin et al. has provided compelling evidence demonstrating that during the course of a year increases in MS relapses show a seasonal preference, particularly for spring (15). These results have recently been confirmed by a large multi-investigator study, which queried International MSBase Registry data pertaining to over 32,000 relapses from >9,000 patients. These data demonstrate that peak relapse risk occurs during spring months regardless of the hemisphere from which the data originated (16).

However, why MS patients exhibit seasonal increases in relapse risk is unknown. Given that vitamin D levels are inversely associated with risk of MS, it is tempting to speculate that subtle changes in UV exposure during different seasons might influence relapse risk. In fact, Spelman et al. have demonstrated a lag in the risk for seasonal influence on exacerbation that is inversely correlated with latitude, indicating that vitamin D generation is inversely associated with relapse risk (16). However, recent meta-analysis data have indicated that vitamin D supplementation does not affect MS relapse (17). Therefore, while vitamin D is associated with disease onset, its role in mitigating exacerbation is currently inconclusive. An alternate hypothesis, and one that is not necessarily mutually exclusive, is that seasonal changes in relapse risk are attributable to viral infection whose incidence is also influenced by seasonality.

### Upper-Respiratory Infection and Relapse Risk

Andersen et al. demonstrated a biannual increase in MS relapse, which was nearly superimposable with the occurrence of the common cold. Notably, an estimated 50–75% of colds are thought to originate from rhinoviral infection of the upper-respiratory system (18). This occurrence has led some to speculate that the majority of MS relapses are a consequence of upper-respiratory infections with members of the *Picornaviridae* family, such as rhinovirus and enterovirus (19, 20). In fact, it has been suggested that upper-respiratory infection by members of *Picornaviridae* accounted for most relapse occurrences (19). However, a subsequent study failed to confirm these findings (21).

That MS relapses are exclusively associated with an infection of a single viral family member, such as rhinovirus, may be farfetched. While rhinovirus infections account for a large percentage of upper-respiratory infections, it should be pointed out that coronavirus, adenovirus, influenza virus, and respiratory syncytial virus are also capable of upper-respiratory infection. In fact, influenza virus infection is also associated with increased relapse risk, a phenomenon, which is mitigated in patients that received previous vaccination (22). Therefore, while it remains uncertain whether particular viruses are more or less associated with MS relapse risk, what is clearly evident is that relapse occurs in an estimated 30–40% of patients subsequent to an upper-respiratory infection (10, 18, 19, 22–24) which has been confirmed by studies employing the use of MRI (24, 25).

Because many pathogens are associated with upper-respiratory infections, it might be anticipated that the biological mechanism underlying their effect on MS relapse is similar, although alterations in viral pathogenesis could account for some discrepancy (see below). In lieu of this hypothesis, it is noteworthy that rhinovirus infections exhibit a biannual peak in incidence, with a small increase in infections occurring during spring months, then the majority of infections occurring during the autumn months (26, 27). Yet data obtained from the meta-analysis by Jin et al. as well as the MSBase Registry clearly demonstrate that the major increase in MS relapse rate occurs during the months that do not completely overlap with the time frame during which rhinoviral incidence would be expected to peak (15, 16). However, the seasonality for all viral infections of the upper-respiratory system is not temporally conserved. For instance, the time frame corresponding to the peak incidence of influenza viral infection, coronavirus, adenovirus, and respiratory syncytial viral infection does not overlap with the time frame of peak rhinoviral infections (28). On the other hand, if the occurrence of any upper-respiratory viral infection contributes to MS relapse risk, it would be anticipated that the increased exacerbation risk time frame would correspond to the cumulative increased incidence in upper-respiratory infections caused by all viruses that infect the upper-respiratory tract. In this regard, it is noteworthy that results from multiple epidemiological studies indicate that over the course of a year, the high-risk time frame for obtaining any upper-respiratory viral infection is very similar to the time frame that corresponds to increased relapse risk in MS patients (15, 16, 28). Moreover, a recent prospective analysis conducted by Tremlett et al. demonstrated that annual MS relapse rates were positively associated with the occurrence of upper-respiratory tract infection and negatively associated with serum 25(OH)D levels (29). Together, these data strongly suggest that the effect of seasonality is correlated with upper-respiratory infection and that upper-respiratory viral infection contributes to relapse risk. Nevertheless, this association still requires experimental validation.

### Viral Reactivation and Relapse Risk

Given the association of viral infections with relapse in MS patients, it is reasonable to hypothesize that the relapsing–­remitting pattern of the initial stages of the disease could perhaps be explained by the dormancy and reactivation of various chronic pandemic viral infections. Particularly, attractive culprits include members of *Herpesviridae* family – including Epstein–Barr virus (EBV), Varicella-Zoster virus (VZV), and human herpes virus (HHV)6. The history of infection with these viruses and MS pathogenesis is extensive. Whether acquired infection with any of these viruses contributes to the onset of disease is not the focus of this review. Rather, here we focus on the possibility that viral reactivation contributes to relapse.

Epstein–Barr virus (Human herpesvirus 4) is a gamma-­herpesvirus exposure to which causes a life-long infection of B-cells that is typically asymptomatic. However, in some cases, the virus causes infectious mononucleosis and in fewer incidences still, lymphoma. Roughly, 90–95% of the population becomes seropositive for EBV antigen by early adulthood. In contrast, nearly all adult MS patients and the majority of pediatric cases have been exposed to EBV (30–32). The intimate association between EBV and MS has sparked great interest in its causal role in the onset of disease. At the very least, the prevalence of EBV in MS patients identifies this virus as a potential cause of infection-induced relapse and thus an agent that is capable of modifying the progression of the disease. However, whether EBV reactivation can promote relapses is uncertain. In fact, only a few studies have presented data that suggest that EBV reactivation promotes MS exacerbation, and most studies on this topic have queried the association between increased EBV-specific serum antibodies and the occurrence of relapse. Collectively, the data indicate that increased circulating EBV-specific antibodies are associated with disease progression and relapse, although few show no association (33). For instance, in one study, a higher percentage of MS patients had antibodies to EBV early antigen when compared to healthy controls, while antibody levels to cytomegalovirus (CMV) were indistinguishable between the groups (34). Interestingly, although there were no differences in anti-EBV early antigen antibody titers between patients in remission versus those undergoing relapse, the presence of these antibodies was associated with enhanced disease activity as determined by MRI (34). However, in a separate study, it was determined that after the first demyelinating event, patients that harbored anti-CMV and/or anti-EBV viral capsid-specific antibodies had a decreased time to relapse when compared with those that did not have detectable levels of these antibodies (35). Similarly, Kvistad et al. have reported that antibodies with a specificity for EBV nuclear antigen 1 (EBNA-1) are also correlated with increased disease activity as determined by MRI, indicating that viral reactivation is associated with relapse (36).

While serum antibody levels to EBV lytic proteins may predict disease progression, they may not be the best tool with which to examine the temporal association between viral reactivation and relapse. The reasons include both a delay in the measurable antibody titer following viral reactivation as well as the relatively long half-life of antibodies in the circulation. A more sensitive approach might be to measure levels of EBV-specific DNA. Capitalizing on this approach, Lindsey et al. investigated the effect of lytic EBV infection on MS activity. Interestingly, the authors did not find differences in serum viral levels between control samples and patients with various types of MS. However, in six patients with measurable viral titers at baseline that had blood drawn before and during a relapse, four showed an increase in virus-specific DNA during relapse (37). Moreover, a recent longitudinal study conducted by the same group provided data, which demonstrated a positive correlation between peripheral blood EBV-specific responses with MRI activity (38). Conversely, the adoptive transfer of *ex vivo* expanded EBV-specific CD8^+^ T-cells into a patient with severe secondary progressive MS has been shown to aid in viral clearance and to reduce disease severity (39). While these preliminary studies currently lack statistical power, these data may indicate that EBV reactivation is associated with relapse for which the virus is either directly or indirectly responsible.

Varicella-zoster virus infection usually occurs early in life and is the cause of chicken pox. Varicella-zoster is neurotropic but for the most part remains dormant in the sensory ganglia. However, immunosuppressive events occurring later in life, such as stress, can result in its reactivation, evidenced by shingles and, in severe cases, encephalitis. Both the neurotropic potential and the relapsing remitting nature of infection make this virus an attractive culprit for influencing the natural history of MS. These traits prompted Graciela et al. to investigate the association of this VZV with MS. Using conventional PCR techniques to screen peripheral blood mononuclear cells (PBMC) for multiple VZV-specific open reading frames, the authors demonstrated a transient increase in viral reactivation in 85% of patients during acute relapse, an effect that was not observed in MS patients during remission (40). These results were repeated in a larger cohort of patients, which found VZV-specific DNA in 38/40 (95%) patients during relapse versus 22/131 (17%) of patients during remission. Using flow cytometry, the authors were also able to show that relapse was associated with an increase of VZV-positive PBMCs when compared to patients in remission or controls (41). In a third study by the same group, the levels of genomic VZV were compared between the cerebrospinal fluid and PBMCs of the same patients. Strikingly, the authors were able to show that VZV viral copy was over 500-fold more abundantly expressed in the CSF compared to PBMCs of patients during relapse. Conversely, VZV was only marginally expressed in the CSF and absent in PBMCs of patients during remission. Furthermore, electron microscopy indicated the presence of VZV viral particles in the CSF of 15 patients during relapse (42). Despite the presence of VZV in both the CSF and PBMCs during relapse, it is notable that there were no changes in serum antibody levels to the virus. Taken together, these studies link VZV reactivation with the occurrence of relapse. Nevertheless, these results should be taken with caution since there has been some difficulty replicating the findings (43, 44).

Like EBV, HHV6 infection usually occurs during childhood and persists for the duration of life; it has also been associated with MS disease pathogenesis (45). In fact, it has been suggested that HHV6 reactivation, as determined by measuring both viremia and anti-HHV specific antibody levels, is strongly correlated with increased disease activity. Moreover, it was demonstrated that HHV infection was 2.5-fold higher for patients with HHV6 reactivation (46). However, results obtained from others indicate a weak association with HHV6 reactivation and exacerbation, as measured by virus-specific cellular immune responses in the blood and MRI activity (38). Others still have provided data suggesting that neither serum HHV6 antibody titers nor viral load is altered between MS patients and control patients (47, 48) and that HHV6 reactivation is not associated with clinical relapse (33).

### Effect of Non-Viral Infections on Relapse Risk

Most research has focused on the association between viral infection of the upper-respiratory system and relapse risk. However, it should be mentioned here that bacterial infections have also been associated with relapse risk. For instance, *Chlamydia pneumonia* infection has also been linked to exacerbation (49). In addition, *Staphylococcus aureus* enterotoxin A has been suggested to be a risk factor for exacerbations, which may indicate that superantigen activation of T-cells is capable of modulating disease (50).

## Animal Models of Infection-Induced Exacerbation

### Overview of the Animal Models That are Used to Study Multiple Sclerosis

To understand the underlying mechanisms that govern infection-induced relapse in MS patients, there needs to be an effective animal model that recapitulates the pathophysiology of human MS. While no animal model achieves this feat, several have been useful tools for investigating different aspects of the disease process. For example, experimental inoculation of genetically susceptible strains of mice with Theiler’s murine encephalomyelitis virus (TMEV) results in a progressive inflammatory-mediated (51–54) demyelinating disease of CNS (55). Likewise, murine infection with neurotropic strains of mouse hepatitis virus can also lead to inflammatory demyelination (56). As such, these viral models are useful in determining the molecular events that are required to overcome tolerance as well as in dissecting the interplay between genetic predisposition and environmental insults.

The most utilized model of human MS by far is experimental autoimmune encephalomyelitis (EAE) and its variants. Typically in this model system, animals are injected with encephalitogenic neuroantigens that have been emulsified in Complete Freund’s Adjuvant (57). Subsequently, animals will develop ascending flaccid paralysis, which is caused by the infiltration and activation of autoreactive T-cells in the CNS parenchyma. The disease can also be passively induced by the injection of activated autoreactive T-cells into naïve animals (58). The use of this model is efficacious for studying the mechanisms governing immune cell trafficking, reactivation, and damage to resident CNS cells (59).

Finally, both *in vitro* and *in vivo* models of remyelination have been established. Remyelination in animals can be assessed using cuprizone intoxication or stereotaxic injection of myelin-degrading agents (lysolecithin and ethidium bromide) (60). In the cuprizone model, mice that are fed the neurotoxicant cuprizone for 3–5 weeks develop consistent demyelination of distinct anatomical regions of the brain (61–63). Demyelination in this model is preceded by oligodendrocyte apoptosis and occurs concurrently with intense reactive gliosis (62–64). However, the blood–brain barrier remains relatively intact (65). Subsequent withdrawal of cuprizone from the diet allows remyelination to occur (66, 67). Both the cuprizone and stereotaxic injection models have proven to be useful in determining the events that control the process of remyelination (60, 68).

### Mechanisms Underlying T-Cell Activation in Response to Infection

**Figure 1:**
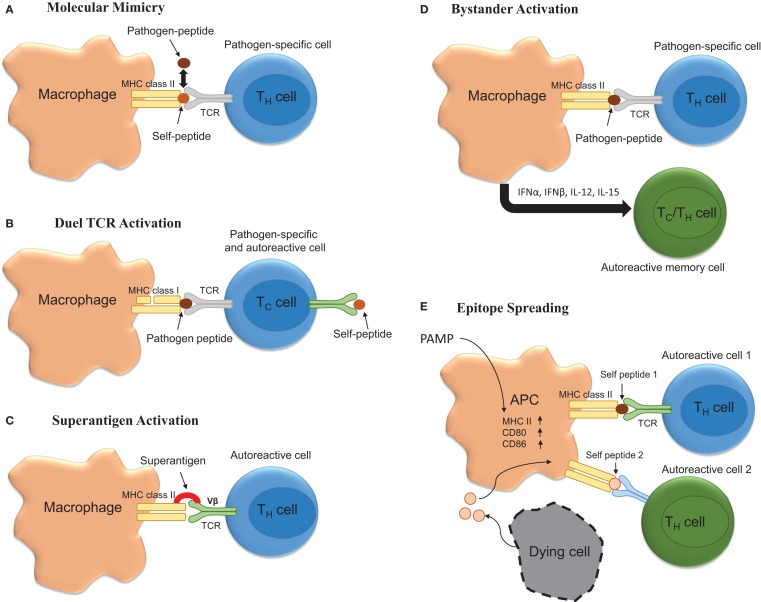
**Potential mechanisms underlying infection induced T-cell activation**. **(A)** Molecular mimicry occurs when there are sufficient overlapping structural similarities between a pathogen-specific peptide and self-peptide such that it triggers T-cell activation. **(B)** Autoimmunity can be triggered by T-cells possessing both pathogen-specific and autoreactive T-cell receptors (TCR). **(C)** Bacterial superantigens can crosslink MHC class II and TCR leading to autoreactive T-cell activation and autoimmune disease onset and/or exacerbation. **(D)** Bystander activation of T-cells by infection could promote exacerbation. Here, infection promotes APC activation leading to the activation of CD44^hi^ polyclonal T-cells via cytokine production. **(E)** Infection via activation of APCs could drive the process of epitope spreading wherein on constitutively released self-peptides resulting from chronic inflammation are presented to polyclonal autoreactive T-cells.

Multiple sclerosis is considered to be a T-cell-mediated autoimmune disease. As such, it is very likely that autoreactive T-cells become activated in response to peripheral infection and that this represents one of the initial events that contribute to relapse. Mechanisms for how autoreactive T-cells become activated in response to infection are described below and are summarized in Figure 1.

Molecular mimicry occurs when pathogen-specific TCRs display cross-reactivity to self-peptides. Importantly, this theoretical frame-work for how self-tolerance is broken and autoimmunity generated and/or perpetuated has been validated in various models of autoimmune disease. For example, C.AL-20 mice infected with herpes simplex virus (HSV)-1 develop herpes stromal keratitis, an autoimmune disease of the eye. Importantly, this disease is recapitulated following the adoptive transfer of T-cells from HSV-1 infected mice to naïve syngeneic *nu/nu* mice (69). Similarly, Olson and colleagues showed that central or peripheral infection of SJL mice with a non-pathogenic strain of TMEV genetically encoding an epitope that resembled one found within the myelin protein proteolipid protein (PLP_139–151_) was sufficient to induce inflammatory demyelinating disease of the spinal cord (70). While TMEV itself did not contain a molecular mimic, these data are important because they show that viruses encoding molecular mimics of self are capable of inducing autoimmunity. Relevant to MS, Wucherpfennig and Strominger showed that myelin basic protein (MBP)-specific T-cell clones isolated from relapsing–remitting MS patients were also capable of recognizing epitopes embedded within several common viruses, including EBV (71). Similarly, it has been shown that microbial antigens derived from *Mycobacterium avium* and *Escherichia coli* are capable of inducing disease in a humanized transgenic mouse model of MS (72). Likewise, peptides derived from *Dictyostelium fasciculatum* and *Emiliania huxleyi* are also capable of inducing EAE in a separate humanized transgenic mouse model of MS (73). Recently, it has become clear that structural mimicry of peptide bound to MHC is a major determinant of this process (72). However, while molecular mimicry is viable hypothesis for how infections can induce MS onset or relapse, several major obstacles impede the establishment of this phenomenon as a key component driving MS pathogenesis. These obstacles include the fact that (1) the autoantigen underlying MS is not known, (2) over time the process of epitope spreading may mask the identity of the molecular mimic (54), and (3) dual TCR expressing CD8^+^ T cells can facilitate disease (74).

Bystander activation represents another means by which peripheral infection could promote MS onset or exacerbation. According to this theory, infection would result in the activation of heterologous T-cells, which have TCR specificity for self epitopes. Bystander activation of T-cells was originally reported to occur in response to peripheral lipopolysaccharide (LPS) and poly(I:C) injections in both CD8^+^ and CD4^+^ T-cell subsets with the former being more susceptible to activation (75, 76). Mechanistically, the cytokines responsible for the bystander effect include IFNα, IFNβ, IFNγ, IL-12, IL-18, and IL-15 (77). As such, this represents a plausible means for explaining how peripheral injections of TLR agonist injections induce disease in TCR transgenic models of EAE.

Another mechanism whereby infection could induce MS relapse is through the release of superantigen. Superantigens act by binding both Vβ regions of the T-cell receptor (TCR) and MHC class II and result in T-cell activation. Indeed, *Staphylococcus aureus* enterotoxin A has been suggested to be a risk factor for exacerbations (50). Moreover, results obtained from studies involving EAE indicate that superantigens can exacerbate or ameliorate disease. Specifically, it has been shown that injection of the superantigen staphylococcal enterotoxin B (SEB) into mice that are in remission from EAE was sufficient to exacerbate disease in 55% of mice compared to 11% of mice receiving an injection of phosphate buffered saline (78, 79). Interestingly, prior infection with a superantigen containing strain of *S. aureus* protected against EAE in a manner that was associated with decreased antigen-specific Th17 responses (80).

Finally, epitope spreading is a process by which chronic inflammation results in the release of self-antigens that are then used to prime self-reactive T-cells. It is likely that peripheral infection, through the activation of antigen-presenting cells perpetuates the process of epitope spreading and facilitates relapse. However, this hypothesis has yet to be thoroughly tested.

### Viral Infections Exacerbate Active EAE

In order to study how peripheral infection can exacerbate MS, some have attempted to model the effect using the various EAE model systems. The results from these studies demonstrate that peripheral infection can exacerbate the pathogenesis of EAE. Of relevance is one study that investigated the role of murine gammaherpesvirus (γHV-68) infection on the pathogenesis of relapsing-remitting EAE in SJL mice. The results demonstrate that infection with live γHV-68 but not UV-inactivated virus exacerbated the disease course. Importantly, this effect was not attributable to viral infection of the CNS and could be recapitulated following adaptive transfer of encephalitogenic MBP-specific T-cells from non-infected animals into animals that were infected with γHV-68 several days prior to transfer (81). However, a follow-up study by Casiraghi et al. found that infection with γHV-68 5 weeks prior to EAE induction was capable of exaggerating the pathogenesis of active EAE in a manner that was independent of viral reactivation but was associated with heightened T-cell (CD4^+^ and CD8^+^) responses within the CNS (82). Notably, it was concluded that γHV-68 was capable of altering antigen-presenting cells in such a way that infection promoted IFNγ production from encephalitogenic T-cells (81, 82). The combined results from both studies indicate that reactivation of herpes viruses may not be required to influence the pathogenesis of EAE. However, the effect of EBV reactivation on the pathogenesis of human MS remains to be determined.

### Effect of Systemic Inflammation on Viral Models of Demyelination

As mentioned, intracerebral inoculation of TMEV into genetically susceptible strains of mice (H-2^s,v,q,r,f^) results in viral persistence and the onset of T-cell-mediated inflammatory demyelination, which ensues approximately 70 days post injection. Conversely, the virus is effectively cleared from the CNS following inoculation of demyelination resistant strains (H-2^d,b,k^), which do not develop demyelination (55). Interestingly, intraperitoneal injection of the TLR4 agonist LPS rendered the prototypical TMEV-induced demyelination-resistant mouse strain (C57BL6) susceptible to inflammatory demyelination. Moreover, the authors demonstrated that injection of recombinant IL-1β was sufficient to recapitulate the results obtained following LPS injection (83). Subsequent studies by the same group suggest that the mechanism whereby peripheral IL-1β promotes demyelination in this normally TMEV-resistant strain entails the generation of a more robust Th17 cell response. However, it should also be noted that the IL-1R1 deficient mice of the same resistant background also became susceptible to demyelination, a paradox, which the authors attribute to the anti-viral properties of IL-1β, which resulted in increased viral persistence within the CNS (84).

### Effect of Systemic Inflammation on Cuprizone-Induced Pathology

As mentioned previously, cuprizone intoxication results in demyelination despite the integrity of the blood–brain barrier’s being maintained (65, 85). Thus, delineating the effect of peripheral infection on either disease progression or remyelination might contribute to understanding of glial physiology during the course of MS. To date, only a few studies have examined the effect of peripheral inflammation on the pathogenesis of disease following cuprizone intoxication. None have yet investigated the effects of peripheral viral infection on the pathogenesis of cuprizone intoxication. As an important first step, it was demonstrated that cuprizone intoxication does not affect the degree of the peripheral acute phase response that occurs subsequent to LPS injection (86). Interestingly, *Il6* was increased in the corpus callosum of LPS-injected mice while *Tnf* and *Il1b* were not significantly affected although this could be attributable to the timing in which the samples were tested (2 h after the final LPS challenge). Remarkably, it was found that repeated peripheral LPS challenge delayed the process of demyelination and promoted remyelination, despite an increase in the number of intralesional RCA-1^+^ microglia. Mechanistically, the effect of LPS was associated with TLR4 upregulation on microglia, increased ciliary neurotrophic factor (*Cntf*) expression, and oligodendrocyte precursor cell proliferation (87). The gp130R ligands CNTF (88–90), leukemia inhibitory factor (LIF) (91–93), and IL-11 (94, 95) are potent inducers of oligodendrocyte proliferation and maturation. As such, these results indicate that in the absence of infiltrating immune cells, peripheral infection-induced glial activation may be beneficial for promoting repair.

### Bacterial Infection Exacerbates EAE

As in viral models of inflammatory demyelination, peripheral LPS injection at 1, 3, and 6 weeks post EAE onset has been shown to cause relapse (96). Aside from LPS injection, other bacterial components and bacteria themselves have also been shown to influence the pathogenesis of inflammatory demyelinating disease. Early studies pertaining to bacterial infection and MS exacerbation focused on the production of superantigenic activation of encephalitogenic T-cells. These studies indicated that peripheral injection of the superantigen SEB was capable of exacerbating relapsing-remitting EAE (79, 97–99). However, it has recently been shown that commensal bacteria are needed to induce EAE (100–102). Moreover, transgenic mice harboring myelin-specific TCR are less susceptible to spontaneous EAE under germ-free conditions (103). Finally, the bacteria-derived toxin, pertussis toxin, is known to lower susceptibility to EAE, and its injection is, in fact, needed to induce disease in some strains of mice (59). However, it is pertinent to mention here that polysaccharide A derived from the capsule of *Bacteroides fragilis* is capable of activating TLR2 on intestinal-derived cells and promotes expansion of Treg cells, decreases Th17 responses, and mitigates EAE onset (100). Likewise, prior infection with a superantigen containing strain of *S. aureus* protected against EAE in a manner that was associated with decreased antigen-specific Th17 responses (80). Together, these studies suggest that systemic inflammation or changes in communalization greatly influence disease susceptibility of classically induced EAE and may point to an underlying theme governing relapses that occur as part of the natural history of MS.

### Genetic Models of EAE and Infection

Nearly 25 years ago, Goverman and colleagues created a transgenic mouse line that expressed a TCR that is specific for MBP. Interestingly, it was noted that mice kept in pathogen-free conditions were impervious to the development of spontaneous EAE, despite the presence of circulating autoreactive cells (103). However, mice maintained in non-pathogen-free conditions spontaneously developed disease between 5 and 23 weeks of age. These results indicate that the exposure to environmental pathogens may provoke autoimmune exacerbation. Along these lines, the authors found that peripheral injection of the Gram-negative bacterial component and TLR4 agonist LPS into mice housed in pathogen-free conditions triggered disease in approximately 60% of mice. Similarly, injection of Complete Freund’s Adjuvant containing heat-killed *Mycobacterium tuberculosis* was sufficient to cause disease in 30% of mice. Finally, peripheral injection of pertussis toxin induced disease in all mice tested (103). Subsequently, Waldner et al. created two transgenic mouse lines (4E3 and 5B6) that harbored a TCR that is specific for the encephalitogenic myelin epitope PLP_139–151_ (104). Both mouse lines were crossed onto the highly EAE susceptible SJL mouse line. Unlike the MBP-specific TCR transgenic mice described above, both the 4E3 and 5B6 mouse lines developed spontaneous EAE despite being housed in specific pathogen-free conditions and being kept on a diet consisting of irradiated food. Interestingly, like the MBP-specific TCR transgenic mouse, injection of pertussis toxin into the PLP-specific TCR line (5B6) readily induced EAE. Furthermore, in a subsequent study, it was noted that the incidence of spontaneous EAE was substantially reduced by 90% when 5B6 mice (on an SJL background) were backcrossed onto the EAE-resistant B10.S background for five generations. Nevertheless, peripheral injections of the TLR9 agonist CpG ODN, the TLR4 agonist LPS, recombinant IL-12, and pertussis toxin were capable of triggering EAE onset (105). Similarly, the transgenic 2D2 mouse line, which possesses an autoreactive TCR to the myelin protein myelin oligodendrocyte glycoprotein (MOG), also exhibits a high incidence of EAE when given pertussis toxin (106). Together, these data indicate that systemic activation of the innate immune response increases the occurrence of spontaneous EAE in transgenic mouse lines that express autoreactive myelin-specific TCR (103–106).

### Peripheral Injection of PAMPs and Lesion Reactivation

Exactly how peripheral infection increases the risk for relapse is not yet known. Mechanistically, infection could promote the antigen stimulation capacity for peripheral antigen-presenting cells (105) including dendritic cells (107), decrease Treg cell function, activate T-cells with dual TCR, activate T-cells via molecular mimicry (71–73, 108), or promote T-cell trafficking and activation to the CNS. Perhaps, there are biological differences between organs that can account for an increased or decreased ability to promote neuroinflammatory responses (109). The above all represent plausible means of contributing to MS relapse and many are a prerequisite for EAE disease induction.

The findings that peripheral injection of particular purified pathogen-associated molecular patterns is independently capable of causing relapse indicate that T-cell activation via cognate antigen recognition may not represent the first step underlying infection-induced relapse (103, 104). To date, several studies have examined the effect of systemic inflammation on exacerbation of EAE. Almost all have found that intraperitoneal inoculation with various pathogen mimics is capable of causing exacerbation to varying degrees.

A few studies have examined the effect of peripheral or central infection on the onset and progression of EAE. While these studies indicate that infection at the time of EAE onset can influence the pathogenesis, delineating a distinct role for infection in facilitating bidirectional communication between the CNS and the peripheral immune system is confounded by the fact that the animal has already received a large dose of heat-killed *M. tuberculosis*. As such, it is difficult, if not impossible, to determine if infection has contributed to the priming of T-cells rather than their attraction and activation within the CNS as would be hypothesized to occur in human cases. In attempts to address this question, several investigators have taken advantage of the fact that Lewis rats develop a monophasic EAE disease course when inoculated with guinea pig spinal cord homogenate in complete Freund’s adjuvant (96). In this model, animals undergo complete remission from disease by day 20 post immunization. Interestingly, peripheral LPS injection 1, 3, or 6 weeks post remission is capable of causing clinical and histological EAE remission (96). Importantly, the authors demonstrate that microglia/macrophage activation was associated with LPS injection and that it preceded disease relapse.

Taking a different approach, Serres et al. have also provided some preclinical evidence that suggests that peripheral infection can reactivate ongoing CNS lesions. In their model system, EAE is induced in rats via subcutaneous injection of neuroantigen emulsified in incomplete Freund’s adjuvant. Next, recombinant TNF and IFNγ are stereotaxically injected into the cingulate gyrus to induce a targeted focal EAE lesion (110). After 4 weeks, the rats receiving an intraperitoneal injection of LPS exhibit lesion reactivation whereas rats injected with a vehicle demonstrate signs of lesion resolution and repair (111). Importantly, these lesions can be followed by MRI, and the lesion status was confirmed by immunohistochemistry. In a follow-up study, the group demonstrated that peripheral injection of an adenovirus encoding IL-1β, but not a control vector, was also capable of reactivating lesions (112).

## Brain-Immune Axis as a Mechanism Underlying Lesion Reactivation and Relapse

### Effectors of Communication Between the Brain and the Immune System

It is conceivable that reactivation of certain neurotropic viruses, such as HSV6 and VSV, would result in microglial activation and subsequent attraction of autoimmune cells to the CNS. However, evidence provided here also indicates that peripheral infection with non-neurotropic viruses or bacteria also have the potential to exacerbate disease in MS patients. Moreover, in transgenic animal models of EAE, peripheral injection of bacterial mimics is sufficient to induce disease, despite an intact blood–brain barrier. These findings indicate that neurotropism is not a prerequisite for disease induction.

Several mechanisms underlying the interaction between cells of the immune system and the brain have been known for decades and have recently been reviewed (113). In brief, peripheral infection upregulates the acute phase proteins IL-1β, TNF, and IL-6. These cytokines are able to be transported across the BBB, to act on endothelial cells to induce inflammatory cytokines that are released basolaterally, to transduce their signal to the CNS via vagal nerve afferent signaling, or to activate cells of the choroid plexus (114). The latter is of particular interest since (1) pathologically MS lesions are typically associated with periventricular white matter destruction (i.e., Dawson’s fingers) (115, 116), and (2) the production of CCL20 by the choroid plexus epithelial cells has recently been reported to serve as a portal for CCR6^+^ encephalitogenic Th17 cells (117, 118).

### Induction of Central Cytokine Expression as a Means for Driving Relapse

**Figure 2:**
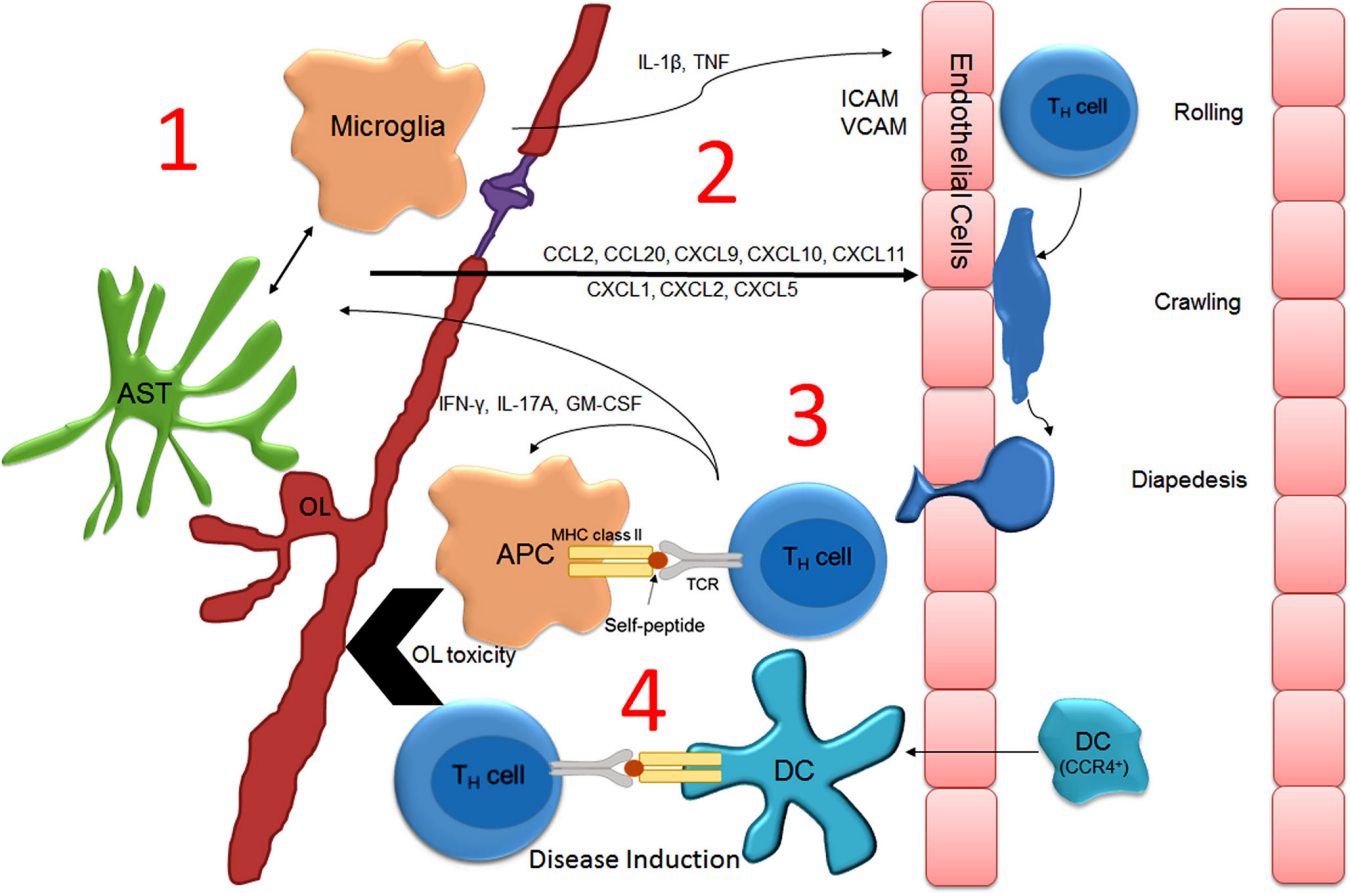
**Model describing how peripheral infection induces neuroinflammation and T-cell-mediated relapse in MS**. During peripheral infection, serum acute phase proteins enter the parenchyma at the choroid plexus, cross the BBB via cytokine transporters, or activate afferent nerves causing glial activation (1). Glial activation induces chemokine (2) and cytokine expression that upregulates endothelial adhesion molecules that promote the extravasation of encephalitogenic T-cell and monocyte/dendritic cell trafficking across the BBB (3). T-cells initially encounter resident perivascular meningeal microglia/macrophages, which have been “primed” for optimal antigen presentation (i.e., upregulation of CD86/CD80 and MHCII). T-cell activation stimulates the recruitment of professional APCs (including CCR4^+^ dendritic cells) and the production of cytotoxic factors, culminating in demyelination and neurodegeneration (4). Abbreviations: APC, antigen-presenting cell; AST, astrocyte; DC, dendritic cell; OL, oligodendrocyte.

That centrally administered recombinant TNF or IFNγ can induce lesion formation in areas of the CNS not normally targeted during the EAE disease course may indicate that cytokine-mediated activation of glia is a prerequisite for the initial attraction of encephalitogenic T-cells to the CNS parenchyma (110). In further support of this hypothesis, Dumas provided convincing evidence demonstrating that IL-1β underlies the effects of pertussis during EAE (119). Intraperitoneal injection of pertussis toxin lowers the activation threshold for EAE induction in many mouse lines and is sufficient to induce EAE in autoreactive transgenic mouse lines. Exactly how peripheral IL-1β or other cytokines promote neuroinflammation is not completely understood. A likely hypothesis is that the production of inflammatory cytokines by infection serves to induce the antigen-presenting capacity of peripheral dendritic cells and B-cells, both of which have been shown to be necessary for the establishment of EAE (107, 120). Nevertheless, microglial activation has also been shown to contribute to the pathogenesis of EAE (121). In fact, a recent temporal analysis focusing on myelin antigen presentation during the course of EAE demonstrated that CNS-resident microglia likely function as the initial antigen-presenting cells as they were shown to contain myelin-associated protein peptides associated with MHC prior to the arrival of peripheral APCs, including dendritic cells (122). Similarly, data obtained from intravital imaging studies employing the use of two-photon microscopy have also challenged the notion that CNS-resident APCs do not present antigen *in vivo*. Specifically, CNS-resident perivascular meningeal cells expressing macrophage but not dendritic cell markers activate extravasated myelin specific but not ovalbumin-specific T-cells (123, 124). Similarly, results from a recent study strongly suggest that microglial-specific deletion of TGFβ-activated kinase 1 (TAK1), a signaling molecule downstream of the IL-1β receptor, ameliorates EAE onset (125). Together, these finding implicate CNS-resident microglial/macrophage activation in driving the initiation of EAE. Notably, IL-1β and TNF are both produced by resident CNS cells in response to peripheral infection and are thus capable of signaling to cells that possess the IL-1β receptor (i.e., microglia and astrocytes) (126–128). In rats challenged by peripheral LPS injection, IL-1β and TNF expression was remarkably increased in the CNS prior to the generation of a relapse (96). Also noteworthy is that stimulation of astrocytes with recombinant IL-1β or TNF in culture dramatically increases chemokine expression. It is therefore plausible that increased chemokine expression within the CNS parenchyma would attract activated T-cells from the periphery into the CNS. Indeed, multiple chemokines have been shown to be required for EAE onset (117, 118) and CCL2, a chemokine that is upregulated in astrocytes after TNF stimulation (129), reportedly sustains immune infiltration after disease onset (130). Given the above evidence, it may be logical to suspect that the same immunophysiological mechanisms that contribute to peripheral infection-induced neuroinflammation and sickness behavior underlie infection-induced relapses. A model for how this might occur is illustrated in Figure 2. Importantly, this model is glial-centric insofar as the activation of glial cells, particularly CNS-resident meningeal antigen-presenting cells and possibly microglia are necessary for the attraction of autoreactive T-cells and professional APCs into the CNS parenchyma. However, while necessary glial activation is not sufficient to promote disease, which has been shown to rely on the antigen-presenting capacity of peripherally derived dendritic cells and B-cells.

An alternative hypothesis, and one that may not be mutually exclusive, is that pulmonary inflammation is somehow linked to autoimmune cell activation and trafficking to the CNS. Evidence supporting this idea includes the fact that smoking, but not the use of other forms of tobacco, is highly associated with MS onset and progression (131–133). Additionally, Odoardi et al. have recently generated compelling evidence, which demonstrates a requirement for encephalitogenic T-cells to enter the lung parenchyma prior to gaining access to the CNS (109). These data indicate that the activation status and/or phenotype of the resident lung antigen-presenting cells may drastically influence the course of MS.

## Conclusion and Future Direction

In conclusion, a great deal of evidence supports a role for peripheral infection in driving MS relapse. This evidence stems from both indirect (seasonal influence) and direct (confirmed viral infection at the time of relapse) experimental animal models. However, the mechanisms by which infection exacerbates the disease course are not fully understood. Uncovering the specific cell signaling pathways that are activated within the CNS in response to peripheral infection may provide some clues as to how infection influences disease progression and would complement what is currently known about how infection induces relapse. Since people in the general population typically acquire one to two upper-respiratory infections per year, understanding the complex biological events that underlie the effect of infection and MS relapse has vast potential for therapeutic intervention and disease mitigation. Moreover, because neuroinflammation is suspected to contribute to the pathophysiology of multiple neurological diseases including but not limited to Parkinson’s disease, Alzheimer’s disease, epilepsy, and depression – the effect of peripheral infection on MS relapse is a highly significant subject for study.

## Conflict of Interest Statement

The author declares that the research was conducted in the absence of any commercial or financial relationships that could be construed as a potential conflict of interest.
